# Do Horses Have a Concept of Person?

**DOI:** 10.1371/journal.pone.0018331

**Published:** 2011-03-30

**Authors:** Carol Sankey, Séverine Henry, Nicolas André, Marie-Annick Richard-Yris, Martine Hausberger

**Affiliations:** Université de Rennes 1, Laboratoire d'éthologie animale et humaine, UMR 6552 CNRS, Station Biologique, Paimpont, France; Université Pierre et Marie Curie, France

## Abstract

**Background:**

Animals' ability for cross-modal recognition has recently received much interest. Captive or domestic animals seem able to perceive cues of human attention and appear to have a multisensory perception of humans.

**Methodology/Principal Findings:**

Here, we used a task where horses have to remain immobile under a vocal order to test whether they are sensitive to the attentional state of the experimenter, but also whether they behave and respond differently to the familiar order when tested by a familiar or an unknown person. Horses' response varied according to the person's attentional state when the order was given by an unknown person: obedience levels were higher when the person giving the order was looking at the horse than when he was not attentive. More interesting is the finding that whatever the condition, horses monitored much more and for longer times the unknown person, as if they were surprised to hear the familiar order given by an unknown voice.

**Conclusion/Significance:**

These results suggest that recognition of humans may lie in a global, integrated, multisensory representation of specific individuals, that includes visual and vocal identity, but also expectations on the individual's behaviour in a familiar situation.

## Introduction

A recent review made a parallel between the human concept of a person, a multisensory percept, and the ability of animals, in particular horses, for cross-modal recognition [1], [2]. Horses were shown to look more rapidly and for a longer time towards a sound source, when the call broadcast was incongruent with the individual seen just before, displaying a response to a violation of expectation [2], as according to Seyfarth and Cheney [1], looking towards a sound source may reveal an expectation of the animal to see the corresponding visual stimulus. Interestingly, dogs also look longer at humans' photographs if they are incongruent with the voice previously heard, which suggests that they had actively generated a visual representation of the auditory information [3]. Dolphins have been shown to look more at familiar humans facing them if they behave in an unexpected manner during a usual task [4]. Captive or domestic animals therefore do have a cross modal perception of humans that involves auditory/visual percepts [3] but more intriguingly still, they seem to also have expectations on their behavior associated with a familiar situation.

Duration of gazes towards humans has also proved useful to evaluate the sensitivity of capuchin monkeys to the attentional state of humans [5], as it is known for human children [6] or infant chimpanzees [7]. They looked more at humans in an eye ceiling situation while pointing an arm to request food, as compared to direct eye contact which may reflect their expectations for humans to look at them in such situations. Could animals too build a rich multisensory concept of person? Could it be that this representation includes identity (*e.g.* voice, face), but also its association with expected behaviours in particular situations?

Dogs obey differently to humans' orders according to the latter's attentional state [8]–[10]. Amongst others, primates, dogs and horses perform differently in a variety of tasks according to a human experimenter's attentional state (eye conditions), all showing a sensitivity to the face/back asymmetry of the human bodies [*e.g.* apes: 11], [ dogs: 9], [12], [ horses: 13].

We hypothesized that horses do, for familiar humans as for conspecifics, integrate multiple cues to form a global representation of an individual. If this was the case, we expected them to have a representation of a familiar person that not only associated voice, face and body features but also expectancies this person may have on their own behaviour. In order to test this hypothesis, we trained young horses to respond to a given order by standing immobile for 60 seconds. Only one person was involved in training, with her face and visual attention always focused on the horse.

If our hypothesis holds, we would expect horses to respond to this same vocal order given by this same person by obeying whatever this person's position and attentional state (facing and looking at the horse, distracted, eyes closed or back turned), while they would be surprised by a novel person giving the same order (violation of expectations) and would be unsure of this person's expectancies. They should therefore increase their monitoring behaviour and be more sensitive to the attentional state of this new person. Therefore, horses were tested with the vocal order given by either the familiar or a novel person, and for each of the 4 situations (facing and looking at the horse, distracted, eyes closed or back turned).

## Methods

Experiments complied with the current French laws related to animal experimentation and were in accordance to the European directive 86/609/CEE. This experiment only included behavioural observations, routine training and non-invasive contacts with the horses (giving food rewards) which did not require the approval of an ethics committee. Permission to carry out the study was given by the COST of the Haras Nationaux. Animal husbandry and care were under management of the staff of the research station in Chamberet, France.

### Subjects

Sixteen two-year old Anglo-Arabian (*N_AA_* = 13) and French Saddlebred (*N_SF_* = 3) horses (*Equus caballus*; 10 females and 6 geldings, *i.e.* castrated males) participated in this study. All horses were born and housed at the 'Station Expérimentale des Haras Nationaux' (SEHN), Chamberet, in France. Four to 6 days after birth (April to June 2007), dams were led with their foals to a large pasture (9 ha) where they stayed all together until weaning at the age of 6 months. They were weaned all together in a pasture in November 2007. They spent the following winter in groups of 5 to 6 individuals in large indoor stalls (∼560 m^2^), where hay was distributed twice a day and water was available *ad libitum*. Concentrated feed pellets were also distributed through automatic devices. They were pushed back to a pasture (7 ha) in the spring 2008. The winter preceding the experiment (November 2008), they were pushed along fenced pathways into individual stalls. These stalls included a large indoor area (4×2 m) and a small outside individual paddock (2×2m). From birth to the beginning of this experiment, human intervention was restricted to food distribution (hay) twice a day in the winter periods and castration and care related to it in males. Apart from this, animals were never handled. Water was always available *ad libitum*.

### Procedure

#### Initial training

In the spring 2009, all subjects underwent a training program to learn to remain immobile in response to a vocal command: “reste!” (*i.e.* French for stay). The whole training was carried out by one and only experimenter (C.S., female). It was the horses' first training experience with a human. The program included successive steps, in which they had to maintain immobility for an increasing duration (from 5 to 60 seconds, cf. **table 1**). Horses had to succeed three times in a row for each step in order to get to the next one and they were positively reinforced using a food reward for each success. The detailed training program can be found in Sankey et al. [14]. Each horse was trained during two daily 5 min sessions for 5 consecutive days, or until they succeeded 3 times consecutively in the last step (*i.e.* immobility for 60 seconds). Training took place in the horses' home stall.

**Table 1:** Description of the steps comprised in the horses' training program.

Steps	Description
Step 1	The horse had to remain immobile for 5 s.
Step 2	The horse had to remain immobile for 10 s.
Step 3	The horse had to remain immobile for 30 s.
Step 4	The horse had to remain immobile for 45 s.
Step 5	The horse had to remain immobile for 1 min.

#### Testing

Horses were tested individually in their home stall (dimensions) and testing started two days after the end of initial training. A video camera was held by an unfamiliar experimenter outside the box and testing was filmed through the bars of the door. The experiment tested whether horses responded differently to the vocal command “reste!” (*i.e*. French for “stay!”) depending on the experimenter's attentional state. Four conditions where visual contact varied were randomly distributed across trials (see further). All conditions were carried out with the familiar experimenter (C.S., woman) and with the unknown person (N.A., man). Testing took place as follows. The experimenter entered the horse's box, placed a halter and attached a lead rope to the horse. She/he placed the lead rope on the horse's neck, looked at the horse and said the horse's name followed by the vocal command “reste!”. Then, the experimenter behaved differently according to 4 experimental conditions, inspired by Call et al. 's study [8]:

#### Looking at condition

The experimenter stood straight facing the horse and looked at its eyes without moving her/his body. If the horse moved its head, she/he tracked the horse's eye with her/his gaze.

#### Eyes closed condition

The experimenter stood straight facing the horse with her/his eyes closed. Her/his body and head orientation were the identical to those in the looking at condition.

#### Distracted condition

The experimenter stood straight facing the horse with her/his eyes looking above the horse, towards the box's ceiling. Her/his body and head orientation were the identical to those in the looking at and eyes closed conditions.

#### Back turned condition

The experimenter turned her/his back to the horse and stood straight looking in front of her/him.

The trial was concluded after 60 s., or as soon as the horse moved before the required 60 s., when the experimenter got a hold of the lead rope and led the horse to its individual outside paddock. At no point during or after the trial did the experimenter react to the horse's actions. That is, she/he neither praised the horse for remaining immobile nor punished the horse for moving. Contrary to training, no reinforcement was used in the testing phase.

Horses were tested twice a day for four consecutive days: on the first two days with the familiar experimenter and on the last two days with the unfamiliar one. For each experimenter, the various conditions were administered in a counterbalanced order to avoid the potential carry-over effects across trials.

### Variables

The following variables were scored for each trial: (a) whether the horse remained immobile for the required 60 s. and (b) the latency to move at least one foot. A maximal latency of 60 s. was attributed to the horses that did not move during the trial.

All occurrences and total time of monitoring behaviour were also recorded [4]. Monitoring was defined as the rotation of the head approximately 45° or more towards the trainer during the time of immobility.

### Statistics

We used a GLM procedure (Minitab 15©) to compare the influence of two factors: person (familiar/unfamiliar) and condition (looking at/distracted/eyes closed/back turned) on horses' monitoring behaviour. The significance of each effect was assessed by considering the F ratio with the highest significant random effect as a denominator for the nested parts of the model. Effects not significant at the α = 0.05 threshold were eliminated. F values for fixed effects were considered to compare the influence of each factor on the results.

We also used non-parametric statistical tests: the Friedman and Wilcoxon signed-ranks tests, to compare matched paired data (*e.g.* the duration of immobility across experimental conditions). An adaptation of the McNemar test, using a binomial test, as suggested by Siegel and Castellan [15], was used to compare success rates.

## Results

**Figure 1:**
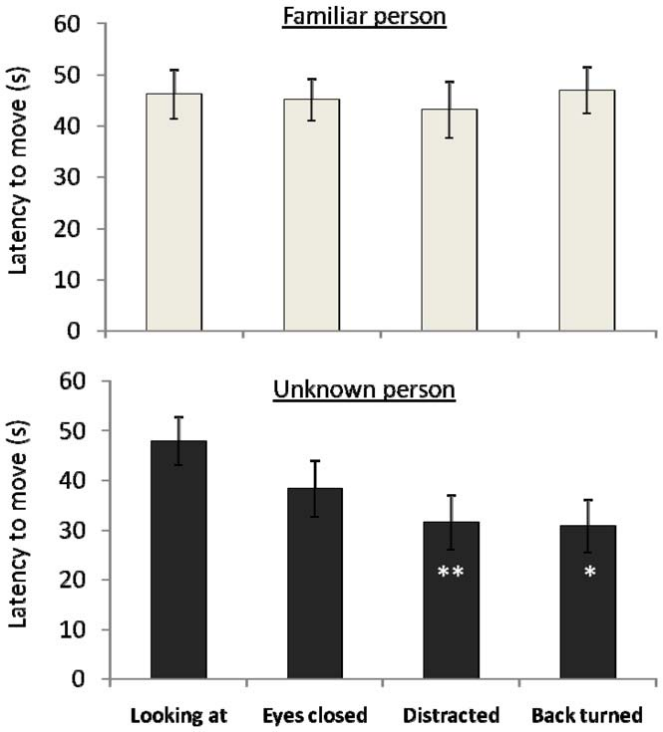
Obedience levels in response to the vocal order: time (s) spent immobile after being given the vocal command “stay!” by the familiar and unknown persons (max: 60 s). Error bars represent standard errors. Wilcoxon *t*-tests, * *P*<0.05, ** *P*<0.01.

**Figure 2:**
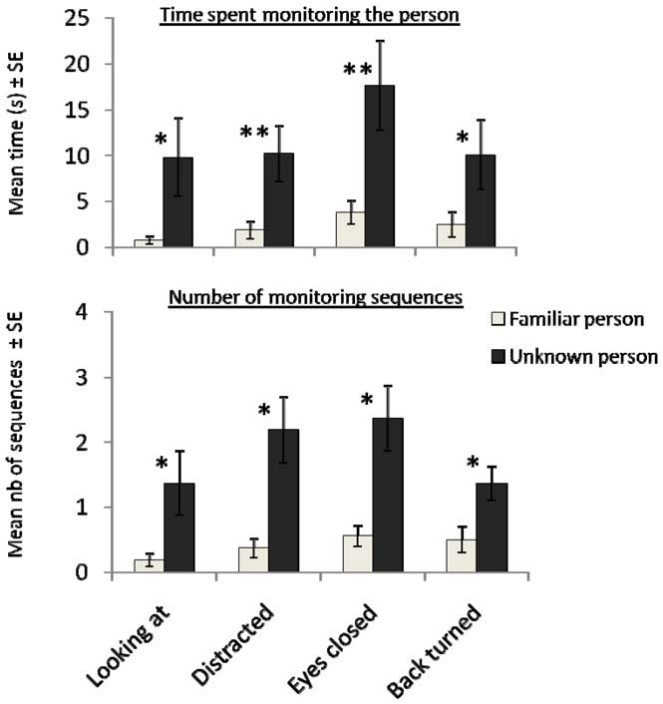
Mean number of monitoring (*i.e.* head rotations of approximately 45° or more towards the trainer during the time of immobility) sequences and mean monitoring duration (s) during the tests in the different conditions with the familiar and the unknown persons. Error bars represent standard errors. * *P*<0.05, ** *P*<0.01.

Clear differences occurred in the level of obedience and its dependency upon the attentional state of the experimenter according to whether she/he was familiar/unfamiliar. Thus, most horses maintained immobility when the familiar trainer looked at them (*N* = 9/16), but also when she turned her back (*N* = 8/16), while the response was slightly lower, though not significantly, for the eyes closed and distracted conditions (*N* = 6/16 for both). The mean time the horses maintained immobility after the order to stay was given did not differ significantly across conditions (**fig. 1**). No differences between conditions were observed in monitoring (*i.e.* head rotation of approximately 45° or more towards the experimenter during the time of immobility) behaviour (**fig. 2**: number of monitoring sequences: Friedman's test, *N* = 16, *P* = 0.31; time spent monitoring the experimenter: Friedman's test, *N* = 16, *P* = 0.25). However, it is interesting to note that most of the monitoring behaviour observed with the familiar person occurred in the “eyes closed” condition.

On the contrary, striking differences appeared according to the attentional state of the unknown person: while most subjects obeyed the order in the “looking at” condition (*N* = 10/16), very few did so in two other conditions (“distracted” condition: *N* = 4/16; “back turned” condition: *N* = 3/16, binomial test *P*<0.05 in both cases). Differences also occurred in the time of immobility (**fig. 1**): subjects maintained immobility for a longer time in the “looking at” (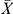 = 47.8±4.8 s) condition than in the “distracted” (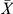 = 31.5±5.4 s, Wilcoxon *t*-test, *N* = 16, *t* = 6, *P* = 0.009) and “back turned” (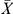 = 30.8±5.3 s, Wilcoxon *t*-test, *N* = 16, *t* = 16, *P* = 0.02) conditions. Intermediate responses were observed for the “eyes closed” condition (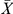 = 38.3±5.7 s). Monitoring behaviour also differed between conditions (Friedman's test, *N* = 16, *P* = 0.04; **fig. 2**) with a clear increase in the “distracted” condition as compared to the “looking at” condition (number of monitoring sequences; Wilcoxon *t*-test, *N* = 16, *t* = 18, *P* = 0.03). Horses clearly responded differently to the familiar and unfamiliar persons: when the person's attention was maximum, obedience levels were similar, but they were clearly lower with the unknown person when he was not attentive.

More interesting is the finding that the effect of the person's familiarity appeared much stronger than the effect of her/his attentional state on horses' monitoring behaviour (GLM: Number of monitoring sequences: F_1–123_ = 21.81, *P*<0.0001; Time spent monitoring: F_1–123_ = 35.35, *P*<0.0001), whereas the “condition” factor did not reveal significant in the GLM procedure (*P*>0.1 for both variables). Whatever the condition, horses showed a higher frequency of monitoring for the unknown person (**fig. 2**: Wilcoxon *t*-tests, *N* = 16, number of head turns: “looking at” condition: *P* = 0.04, *t* = 5; “eyes closed” condition: *P* = 0.03, *t* = 3.5; “distracted” condition: *P* = 0.03, *t* = 3; “back turned” condition: *P* = 0.02, *t* = 12.5) and monitored him for much longer durations (**fig. 2:** Wilcoxon *t*-tests, *N* = 16, time monitoring: “looking at” condition: *P* = 0.04, *t* = 7; “eyes closed” condition: *P* = 0.01, *t* = 13; “distracted” condition: *P* = 0.006, *t* = 4; “back turned” condition: *P* = 0.02, *t* = 16.5). It is also interesting to note that the highest level of monitoring the familiar person was observed in the eyes closed condition, even though this was not significant (**fig. 2**).

## Discussion

In response to a known vocal order, horses obeyed similarly to attentive (with eye contact) familiar and unfamiliar persons but they monitored much more the stranger's behaviour by turning their head and gazing at him. This was even more the case when the attentional state of the person appeared lower, by turning his back, closing his eyes or looking above the horse (distracted).

These results suggest high representation levels, based on sophisticated interspecific socio-cognitive skills. In our study, horses seemed surprised to hear the familiar order given by an unknown voice, as shown by their increased monitoring behaviour (violation of expectations). It appears as if they were trying to identify the person's intentions or expectations, by monitoring his visual attention, for the eyes carry a great deal of information on one's attentional state [16]. Here, we observed disturbances in the horses' response when the experimenter's eyes were not visible or when they were not directed at the horse: they disobeyed the command given by the novel person more readily when he was distracted and when he had his back turned than when he was looking at them. Whereas the back turned seems to be a fairly easily identifiable state of inattention in many species [*e.g.* dogs: 9], [12]; [ apes: 11], horses, like dolphins, have the specificity to have laterally placed eyes that allow them to have a very large visual field covering almost the 360° around them [17]. Nevertheless, they often prefer facing a human when monitoring him and seem able to understand the asymmetry of humans' front and back sides.

Laboratory and field experiments conducted with baboons suggest that the memory of recent interactions with particular individuals determine whether they judge a particular vocalization as directed to them or not [18], [19]. Having no previous experience with the unknown experimenter, horses may have here wondered whether the order was or was not directed to them.

Horses's obedience and behaviour were very different with the familiar trainer: their obedience to the order did not differ across conditions and their monitoring behavior was low and unaffected by the experimenter's attentional state. In fact, they also obeyed when she had her back turned. One explanation could be that they knew this trainer very well and had developed with her a relationship that allowed them to anticipate her expectations, whatever the environmental disturbances. This would well illustrate Hinde's definition of a relationship [20] that states that once a relationship is established, partners have expectations on the other's behaviour and the issue of the following encounters can therefore be foreseen. These expectations are based on the previous interactions. Given the training history of our horses with their familiar trainer, they might have learnt to know her and her expectations, therefore responding similarly to her order whatever her attentional state, even when she had her back turned. It was actually the “eyes closed” condition that elicited, though not significantly, the most monitoring behaviour. If horses do have a representation of a person based on experience, this is not very surprising, as they are bound to have seen the experimenter with her back turned or distracted at some point during the training sessions, while seeing her with her eyes closed was something completely new.

Supported by the recent literature [1], [2], these results suggest that animals' recognition of others may lie in a global, integrated, multisensory representation of specific individuals, that includes visual and vocal identity, but also both their expectations on the individual's behaviour in a familiar situation and this individual's expectations on their own behaviour. In humans, we would call this representation the “concept of person”, which might be more widespread in domestic animals than we once thought.
